# Calcium Glycerolate Catalyst Derived from Eggshell Waste for Cyclopentadecanolide Synthesis

**DOI:** 10.3389/fchem.2021.770247

**Published:** 2021-12-09

**Authors:** Haijun Cheng, Jiangli Wei, Min Liang, Suyi Dai, Xiongmin Liu, Li Ma, Hongyun Wang, Fang Lai

**Affiliations:** School of Chemistry and Chemical Engineering, Guangxi University, Nanning, China

**Keywords:** eggshell waste, low-temperature calcination, calcium glycerolate, macrolactonization, cyclopentadecanolide

## Abstract

**Abstract:** The synthesis costs of macrolide musks are higher than those of other commercial musks. To make this process less expensive, eggshell waste was calcined at a low temperature to obtain a catalyst for the cyclopentadecanolide production *via* reactive distillation using a glycerol entrainer. X-ray diffraction, Fourier-transform infrared spectroscopy, scanning electron microscopy, transmission electron microscopy, and X-ray photoelectron spectroscopy analyses of the original and recovered catalysts revealed that the main catalytic ingredient was calcium glycerolate (CaG) and not calcium diglyceroxide (CaDG). The basic strengths of CaG and CaDG obtained by Hammett indicators were 7.2 < H_≤ 15.0 and 9.8 < H_≤15.0, while the corresponding base amounts were 1.9 and 7.3 mmol/ g, respectively. Because CaG was soluble in glycerine, the catalyst was efficiently reused. The reaction product containing over 95.0% cyclopentadecanolide with a yield of 49.8% was obtained at a temperature of 190°C and catalyst amount of 12 wt% after 7 h of reaction. Thus, eggshell waste may be directly placed into the reaction mixture after calcination at 600°C to synthesise a large amount of cyclopentadecanolide within a relatively short time. The results of this work indicate that eggshell waste can serve as a potential eco-friendly and affordable catalyst source for the production of macrolide musks.

## Introduction

Eggs are consumed worldwide due to their high nutrition value. Eggshells constitute approximately one-tenth of the egg mass (Christmas and Harms, 1976) and are typically discarded as kitchen or solid waste without pretreatment (Kamkum et al., 2015). In China, several million tonnes of eggshells are generated annually, and this amount will continue to increase in the future. During the eggshell degradation process, hydrogen sulphide and other toxic gases toxic are generated to become a source of organic pollution (Oliveira et al., 2013). Hence, it is not desirable to landfill eggshell waste directly due to environmental concerns. Instead, eggshell waste can be potentially reused to eliminate the environmental threat and produce a high economic value (Xu et al., 2019; Mohazzab et al., 2020; Punj and Singh, 2020).

Eggshells comprise a network of protein fibres, in which calcium carbonate accounts for 96% of the shell weight, the contents of calcium phosphate and magnesium carbonate are 1% each, and the remaining part consists of organic matter and water. Because limestone is a non-renewable natural resource, using renewable eggshell waste as an alternative source of calcium carbonate can reduce the negative impact on the natural reserves of limestone. Calcium carbonate may be converted into calcium oxide by calcination, and the calcium oxide prepared from eggshell waste can potentially replace more expensive commercial calcium oxide reagents. The pores of eggshells may be used as catalyst carriers (Nasrollahzadeh et al., 2016), and the calcium oxide obtained after calcination represents a high-quality base catalyst (Verziu et al., 2011; Marinković et al., 2016). Therefore, developing eggshell catalysts provides a reliable direction for the effective use of eggshells waste. Eggshell-derived catalysts have been successfully applied in biodiesel production (Joshi et al., 2015; Yusuff et al., 2018; Kirubakaran and Arul Mozhi Selvan, 2018; Marwaha et al., 2018; Rahman et al., 2019). Wei et al. (Wei et al., 2009) obtained a highly active reusable solid catalyst by calcining eggshells and utilised it to convert soybean oil into biodiesel. When the calcination temperature of eggshells was below 600°C, the biodiesel yield was less than 30%, and its value was increased by raising the calcination temperature to 1,000°C. Similarly, eggshells and chicken fat constitute poultry wastes that can be valorised as cheap sources of the catalyst and oil for the production of biodiesel, respectively. Heterogeneous catalysts were successfully prepared by calcining eggshells, and the maximum biodiesel yield of 90.2% was achieved at a calcination temperature of 1,000°C (Odetoye et al., 2021).

In addition to catalysing the synthesis of biodiesel, eggshell-derived calcium oxide has also been employed in other catalytic reactions. (Gao and Xu, 2012) utilised eggshell waste as a raw material to prepare a catalyst for the synthesis of dimethyl carbonate (DMC). Eggshells were calcined in a muffle furnace at 1,000°C for 2 h under static air prior to the reaction. The obtained DMC yield was as high as 75%. Taufiq-Yap et al. (Taufiq-Yap et al., 2014) studied the possibility of hydrogen production via wood gasification facilitated by an eggshell-based catalyst. The biomass gasification of Azadirachta excelsa wood was performed using a CaO catalyst derived from the eggshells calcined at 900°C for 2 h. As a result, the hydrogen production was increased to a maximum value of 73%. Khazaei et al. (Khazaei A. et al., 2017; Khazaei et al., 2017b) employed a CaO catalyst derived from eggshells as a natural solid base for the Suzuki coupling reaction conducted in the presence of rice husk-supported Fe_3_O_4_ that stabilised palladium magnetic nanoparticles. The eggshell calcination temperature in that study was 900°C. A CaO nanocatalyst prepared from hen eggshells was used for the production of pyrano [4,3-b]pyrans. Eggshell powder was calcined at 900°C for 1 h, and the obtained product exhibited high catalytic activity (Mosaddegh and Hassankhani, 2014). In addition, eggshell waste can be reused multiple times in various catalytic processes, demonstrating very high stability. In the production of biodiesel and other industrial compounds, not only high yield and high value-added products were obtained, but the use value of eggshell waste was also improved, which allowed the successful reuse of this waste, decreased the production costs, and reduced environmental pollution.

Eggshell-derived calcium oxide is utilised as a catalyst, and its active ingredients depend on a particular catalytic system (Kouzu et al., 2008; Ebadi Pour et al., 2021). Specifically, when calcium oxide is extracted from eggshells as a catalyst to produce biodiesel, the obtained product contains not only calcium oxide, but other components as well. Furthermore, calcium oxide is easily converted into calcium glyceroxide (CaDG) through its reaction with glycerol. Similar to calcium oxide, CaDG effectively catalyses the transesterification of vegetable oil (Kouzu et al., 2010). Gupta’s group used eggshell waste to derive two catalysts: Eggshell–CaO_C–H–D_ and Eggshell–CaDG, which were subsequently used for the transesterification of waste cooking oil The obtained biodiesel yields were 96.07 and 93.10%, respectively (Gupta and Rathod, 2018b). Therefore, further research is required for the catalytic utilisation of calcium oxide derived from eggshells. In general, eggshells are utilised in many fields, and their catalytic efficiency is very high. However, when calcium oxide derived from eggshells is used as a catalyst, the calcination temperature of eggshells in the above-mentioned reactions reached 900 °C and even exceeded 1,000°C in some cases, which resulted in very large energy consumption. Thus, our objective was to identify a synthetic reaction catalysed by a solid base which was obtained by the low-temperature calcination of eggshells. To the best of our knowledge, no studies on the catalytic synthesis of macrocyclic musks using eggshell derivatives have been performed previously.

The very special olfactory feeling exerted by musk or ‘muskiness’ is generally described as a unique blend of softness, gentle warmness, and sensuality with a skin-like impression (David, 2020). A well-known and best-selling class of fragrance compounds includes macrocyclic lactones and ketones known for centuries as musks (Sytniczuk et al., 2018). In the past, musks were obtained from fauna and flora leading to a severe decrease in their population and bringing many species to the verge of extinction. Therefore, people have turned their attention to synthetic musks, leading to significant achievements in this field (Nagarajan et al., 1999; Zviely, 2009; Ohloff and Pickenhagen, 2012; Parenty et al., 2013; Li et al., 2017; Zhang et al., 2018). These synthetic musks are widely used in many personal care products, including soaps, shampoos, detergents, and deodorants as well as in scented products such as air fresheners and scented candles (Belsito et al., 2011). Other musk applications include antibiotics (Jelić and Antolović, 2016), anti-cancer drugs (Luesch et al., 2002; Wright et al., 2007; Fuwa et al., 2011), food additives, and insecticides (Kirst, 2010; Bai et al., 2016). In 2017, the estimated market for flavours and fragrances was worth 24.8 billion dollars (International Flavors and Fragrances Inc., 2017). Macrocyclic musks have attracted increasing attention from the perfume industry (De Léséleuc et al., 2015) as they do not exhibit toxicity or bioaccumulation properties associated with the traditional nitro-aromatic and polycyclic musks (Zviely, 2009). In addition, they are environmentally friendly, have renewable alternatives, and possess good sustainability properties (Morin et al., 2019). The market demand for macrocyclic musks is rapidly growing (Matsuda et al., 2007; Boethling, 2011). Synthetic macrolactones, which represent an alternative to those found in natural sources (Blunt et al., 2009), have also attracted considerable attention from researchers (Morales-Serna et al., 2010). Cyclopentadecanolide is a semi-volatile cyclic organic chemical, which belongs to the class of macrolide musks. An important strategy for the synthesis of macrolide musks is the macrolactonisation of seco acid, which has been performed in a large number of synthetic processes. In 1998, the lactonisation of 15-hydroxypentadecanoic acid to cyclopentadecanolide was successfully conducted on dealuminated HY zeolite (Ookoshi and Onaka, 1998). The macrolactonization reaction utilises m-xylene as a solvent to obtain a 51% yield of cyclopentadecanol after 24 h reflux at a very low raw material concentration. Myléne de Léséleuc et al. (De Léséleuc and Collins, 2015) used Hf(OTf)_4_ to directly catalyse the macrolactonisation of seco acid *via* a ‘one-pot’ reaction. However, this method has several inherent limitations. For example, either the acid or hydroxyl groups must be activated by special reagents. In addition, to minimise intermolecular dimerisation, highly diluted conditions or slow addition protocols are required.

Zhang et al.(Zhang et al., 2018) used Rh(I)/Yb(III) to catalyse the macrolactonisation of alkynyl alcohol and provide a strategically distinct entry for macrolactones. In addition to the operational simplicity, this process can be performed at relatively high concentrations. Although many catalysed macrolactonisation reactions were conducted in the past (Gonthier and Breinbauer, 2005; Kopp et al., 2012; Parenty et al., 2013; Li et al., 2017; Xie et al., 2018), their practical implementation remains challenging due to the complex catalyst preparation process, large macrolactonisation time, high production costs, and the difficulty of obtaining intermediates. Furthermore, the reaction product must undergo complex separation and purification processes requiring large amounts of organic solvents, and the final macrolactone yield is very low. Hence, the synthesis of macrocyclic musk compounds is a difficult and, in many cases, multi-step procedure.

In this work, we studied the catalytic properties of an eggshell-derived catalyst for the macrolactonisation of methyl 15-hydroxypentadecanoate to cyclopentadecanolide using a reactive distillation method. Because both the reactant and the product are insoluble in glycerol, the latter is employed as an entrainer during the distillation of the raw materials for the synthesis of catalytically active compounds. The obtained catalyst was characterised by X-ray diffraction (XRD), thermogravimetric analysis (TGA), Fourier-transform infrared (FT–IR) spectroscopy, and scanning electron microscopy (SEM). The yields of the produced cyclopentadecanolide were determined by gas chromatography (GC) and optimised under various operating conditions, including calcination temperature, reaction temperature, reaction time, and catalyst amount. As a result, eggshell waste was found to be an economical and eco-friendly catalyst for the synthesis of macrocyclic musks.

## Materials and Methods

### Chemicals

Chicken eggshells were collected from a canteen at Guangxi University located in Nanning City, China. Diethyl ether, methanol, ethanol, sulfuric acid, n-hexane, bromothymol blue, phenolphthalein, 2,4,6-trinitroaniline, 4–nitroaniline, and glycerine was purchased from Guangdong Guanghua Sci-Tech Co., Ltd. (Guangdong, China). Cyclopentadecanolide (>98%) was obtained from Sigma-Aldrich Co., and 2-amino-2-methyl-1-propanol and calcium oxide were purchased from Shanghai Aladdin Biochemical Technology Co., Ltd. (Shanghai, China). All materials were of analytically pure grade. Malania Oleifera Chum oil was obtained from Yandong Township, Bama County, Guangxi Province, China.

### Catalyst Preparation

The collected eggshells were rinsed with distilled water to remove impurities. The washed eggshells were dried overnight at 110°C and then crushed into powder using a mortar, which was subsequently calcined in a muffle furnace for 4 h. Before cooling to room temperature, the calcined sample was transferred into a sealed glass container.

CaDG was prepared as described elsewhere (Kouzu et al., 2010; León-Reina et al., 2013; Gupta et al., 2015; Esipovich et al., 2018). Briefly, 0.5 g of CaO was directly placed into a flask containing 35 ml of glycerol and 100 ml of methanol. The obtained mixture was stirred at a speed of 600 rpm and temperature of 80°C for 10 h. The obtained precipitate was separated by centrifugation and washed with methanol until the unreacted glycerol was completely removed and dried at 95°C for 2 h.

CaG synthesis was performed according to the method developed by the Taylor group (Whitehouse, 1992). In this process, 3 g of calcium hydroxide was added to 30 g of glycerol, and the obtained mixture was heated to 180°C for 4 h. After cooling to room temperature, the solids were washed with 40 ml of anhydrous ethanol solution and centrifuged at 3,000 rpm. After repeated washing, the collected solids were dried overnight in vacuum at 60°C.

### Catalytic Test

#### Synthesis of Methyl 15-Hydroxypentadecanoate

15-Tetracosenoic acid was obtained from *Malania Oleifera Chum* oil after saponification, acidification, and solvent crystallisation. 15-Tetracosenoic acid (30.0 g) was dissolved in a n-hexane/methanol(4:1, 200 ml) mixture. The entire reactor assembly was immersed into a thermostatic water bath with a constant temperature of 0°C. Ozone gas generated by a laboratory-scale corona discharge generator was continuously bubbled through the reactor until reaching a turning point, at which potassium iodide starch paper turned blue. Potassium borohydride solution which was prepared by dissolving 0.2 g NaOH and 5.5 g KBH_4_ in 180 ml of deionised water was slowly dropped into the obtained ozonide intermediate at a temperature between 15 and 18°C and continuously stirred for 3 h. The pH value of the produced alkali liquor was adjusted to 2 by adding a 6 mol/L hydrochloric acid solution. The mixture was decompression filtered, and the 15-hydroxypentadecanoic acid filter cake was washed to the neutral pH by distilled water and dried at 70°C for 8 h under vacuum to remove water and other solvents.

The obtained 15-hydroxypentadecanoic acid was purified by forming an amine compound with 2-amino-2-methyl-1-propanol. After that, methyl 15-hydroxypentadecanoate was produced by methyl esterification. In this process, 15 g of 15-hydroxypentadecanoic acid was dissolved in 300 ml of methanol followed by the slow addition of 2 ml concentrated sulfuric acid and refluxing at 90°C for 4 h. After the reaction was complete, the product was extracted three times with diethyl ether and washed with water. The solvent was removed by rotary evaporation and concentrated to obtain methyl 15-hydroxypentadecanoate with a mass content of more than 70%.

### Synthesis of Cyclopentadecanolide

Cyclopentadecanolide (2) was synthesised as follows. First, 0.5 g of the catalyst and 5 mmol of methyl 15-hydroxypentadecanoate (1) were mixed with 15 ml of glycerine solution and stirred at 120°C for 30 min until methyl 15-hydroxypentadecanoate was completely dissolved. Afterwards, the solvent and cyclopentadecanolide were distilled out from the reaction system at 190°C in vacuum (2 mbar), and the solvent was returned to the reaction medium. At the end of the reaction, the system was cooled to room temperature (25°C). The produced cyclopentadecanolide crystals were taken out and dissolved in 10 ml of ether and washed three times with 50 ml of deionised water. The upper solution layer was separated, and the solvent and water were removed under vacuum at 60°C. Finally, a white lumpish solid (cyclopentadecanolide) was obtained.

### Catalyst Characterisation

XRD patterns of the produced samples were recorded on a Rigaku Ultima IV diffractometer with Cu Kalpha radiation in the 2*θ* range of 5–80°.

TGA and derivative thermogravimetry (DTG) studies were performed using a TA Instruments Q500 analyser at a heating rate of 10°C min^−1^ in the temperature region from 30 to 1,000°C under a nitrogen atmosphere.

XPS measurements were conducted on a Thermo Scientific K-Alpha spectrophotometer.

Morphologies and chemical compositions of the obtained catalysts were examined using a scanning electron microscope equipped with an energy dispersive X-ray analysis (EDX) module (Zeiss Sigma 300, SmartEDX).

FT–IR spectra were recorded on a Nicolet iS50 spectrometer in the wavenumber range of 4,000–800 cm^−1^ using KBr pellets.

Hammett indicator (dissolved in ethanol) was utilised to determine the basic strengths of the catalysts (Rahman et al., 2019). Briefly, 0.1 g of the analysed sample was shaken with 10 ml of moisture-free ethanolic solution of the Hammett indicator and left to equilibrate for 2 h. The indicators used were bromothymol blue (pK_a_ = 7.2), phenolphthalein (pK_a_ = 9.8), 2,4,6-trinitroaniline (pK_a_ = 12.2), 2,4–dinitroaniline (pK_a_ = 15.0), and 4–nitroaniline (pK_a_ = 18.4). Afterwards, 2–3 drops of the Hammett indicator solution were added to an ethanolic solution of the sample with gentle shaking and then left to be equilibrated for colour variation. If the indicator exhibited a colour change, the basic strength of the catalyst was considered to be higher than that of the indicator; otherwise, the catalyst basic strength was lower than that of the indicator. Catalyst basicity was assessed by measuring the amount of the Hammett indicator–benzene carboxylic acid solution required for its neutralisation (Yan et al., 2009).

### Product Analysis

GC analyses of the synthesised methyl 15-hydroxypentadecanoate and cyclopentadecanolide products were performed on a Shimadzu GC-2010 Plus gas chromatograph. Their yields were determined *via* the following formula:

$$yield\left(\%\right)=\frac{m\left(product\right)}{m\left(theory\right)}\times 100\%$$

## Results and Discussion

### Catalytic Performance

#### XRD Analysis of Eggshell Waste at Different Calcination Temperatures

**FIGURE 1:**
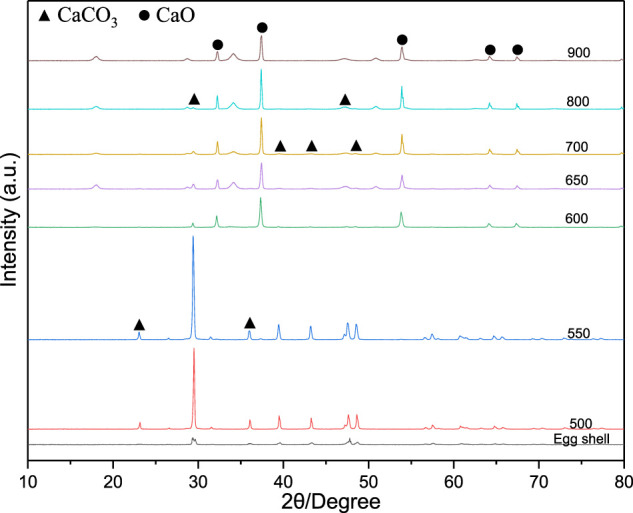
X-ray diffractograms of the calcined eggshells.

The XRD patterns of the eggshells calcined at different temperatures are shown in Figure 1. The untreated eggshell spectrum contains a weak CaCO_3_ (PDF#47-1743) diffraction peak. In contrast, the XRD patterns of the eggshells calcined at 500 and 550 °C had very intense and sharp CaCO_3_ peaks centred at 23.06°, 29.40°, 35.97°, 39.41°, 43.16°, 47.50°, and 48.50° (PDF#72-1214), owing to the removal of organic impurities from the eggshell surface. When the calcination temperature exceeds 600 °C, the eggshells exhibit pronounced CaO diffraction peaks at 32.19°, 37.34°, 53.84°, 64.13°, and 67.36° (PDF#77-2010). As the calcination temperature further increases, the CaO diffraction peak gradually increases, and the CaCO_3_ diffraction peak gradually decreases until it completely disappears at 900°C because the CaCO_3_ phase in the eggshell has been fully converted into CaO. Therefore, the minimum temperature at which eggshell waste is converted into calcium oxide is 600°C.

#### TG/DTG Analysis of Eggshell Waste

**FIGURE 2:**
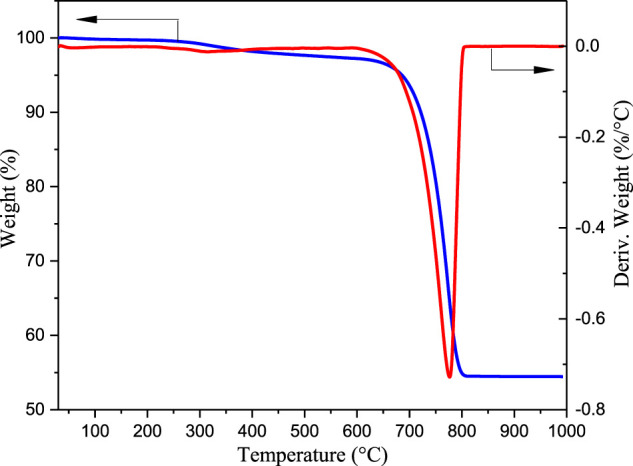
TG/DTG curves of the calcined eggshells.

The calcination process of waste eggshell powder was studied by TG/DTG, and the obtained results are shown in Figure 2. The eggshell powder was heated from 30 to 1,000 °C at a rate of 10°C min^−1^ under a nitrogen atmosphere. The quality reduction of eggshell powder observed at 30–500°C was mainly caused by the decomposition of organic matter and moisture removal from the eggshells. The quality of eggshell powder at 600–800°C was significantly reduced due to the removal of carbon dioxide. However, the eggshell quality changed very little after 850 °C, indicating that CaCO_3_ was completely decomposed into CaO at this temperature. From the results of TG/DTG analysis, it can be concluded that the temperature at which CaO starts to produce and CaCO_3_ begins to decompose is 600 °C consistent with the XRD data. Therefore, considering the preparation temperature and energy consumption, the CaO catalyst can be obtained from waste eggshells by roasting them at 600 °C. Similar results were obtained for waste eggshells by Joshi et al.(Joshi et al., 2015).

#### FT–IR and XRD Analyses of Different Catalysts

**FIGURE 3:**
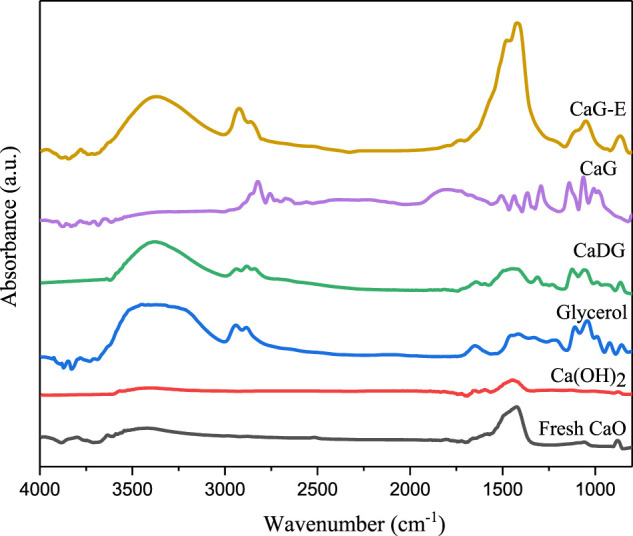
FT–IR spectra of various solids and glycerol.

The FT–IR spectra recorded for fresh CaO derived from waste eggshells at 600 °C, Ca(OH)_2_, CaDG, CaG, CaG-E (used catalyst obtained from CaO derived from eggshells), and glycerol are presented in Figure 3. All samples are characterised by a broad band between 3,600 and 3,000 cm^−1^ which can be ascribed to O–H stretching vibrations. In addition, the FTIR spectra of fresh CaO exhibit O–H stretching vibrations, which were likely caused by the absorbed water from air. The bands at 2,925, 2,865, and 2,826 cm^−1^ in the CaDG, CaG, CaG-E, and glycerol spectra are related to C–H stretching vibrations. Similarly, various C–H bending modes (1,436, 1,228, and 921 cm^−1^), C–O–H bending modes (1,311 cm^−1^), and C–O stretching modes (1,104 and 1,050 cm^−1^) were observed. The band between 1,300 and 1700 cm^−1^ corresponds to the O–C–O stretching vibrations of CO_3_^2−^ species. The FT–IR spectra of CaDG, CaG, and glycerol are in good agreement with the results reported by Reyero et al.(Reyero et al., 2014) and León-Reina et al.(León-Reina et al., 2013). They show that CaDG and CaG have been successfully synthesised and used in the cyclopentadecanolide production. Note that the FT–IR spectrum of CaG-E is similar to those CaDG and CaG and exhibits a high O–C–O stretching vibration intensity, owing to the loss of CaG from the solid catalyst surface and exposure of CaCO_3_ species.

**FIGURE 4:**
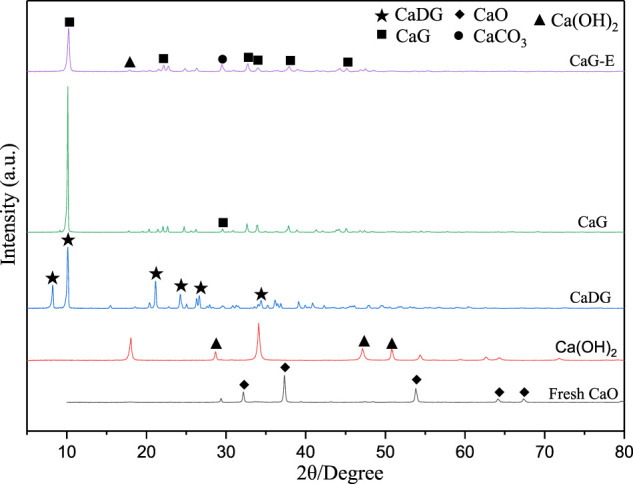
X-ray diffractogram of various solids.

The XRD patterns of fresh CaO, Ca(OH)_2_ (2*θ* = 18.01°, 28.67°, 34.10°, 47.12°, 50.81°, PDF#44-1,481), CaDG (2*θ* = 8.18°, 10.09°, 21.19°, 24.30°, 26.59°, 34.37°, PDF#21-1,544), CaG (2*θ* = 10.13°, 22.08°, 29.56°, 32.62°, 33.92°, 37.84°, 45.06°), and CaG-E are presented in Figure 4. The CaG-E spectrum contains characteristic diffraction peaks of CaG (2*θ* = 10.13°, 22.08°, 32.62°, 33.92°, 37.84°, 45.06°, Taylor et al. (Whitehouse, 1992)) and Ca(OH)_2_ (2*θ* = 18.01°, 34.10°) with lower intensities, suggesting that CaG and Ca(OH)_2_ were formed in the reaction of CaO with glycerol at high temperatures, which was consistent with the results obtained by Pour et al. (Pour et al., 2021). Moreover, colloidal Ca(OH)_2_ was formed due to the presence of water in the reaction medium (Ruppert et al., 2008), and when CaO and Ca(OH)_2_ were converted *in situ* to solid CaG, an equilibrium was established between CaG and colloidal Ca(OH)_2_ species. The diffraction peak of CaCO_3_ (2*θ* = 29.18°) in the CaG-E spectrum is more pronounced, which is related to the loss of surface Ca(OH)_2_ species during the washing recovery process. Hence, CaG is the active component of the produced catalyst, which is in good agreement with the FTIR data.

#### SEM and EDX Analyses of Different Catalysts

**FIGURE 5:**
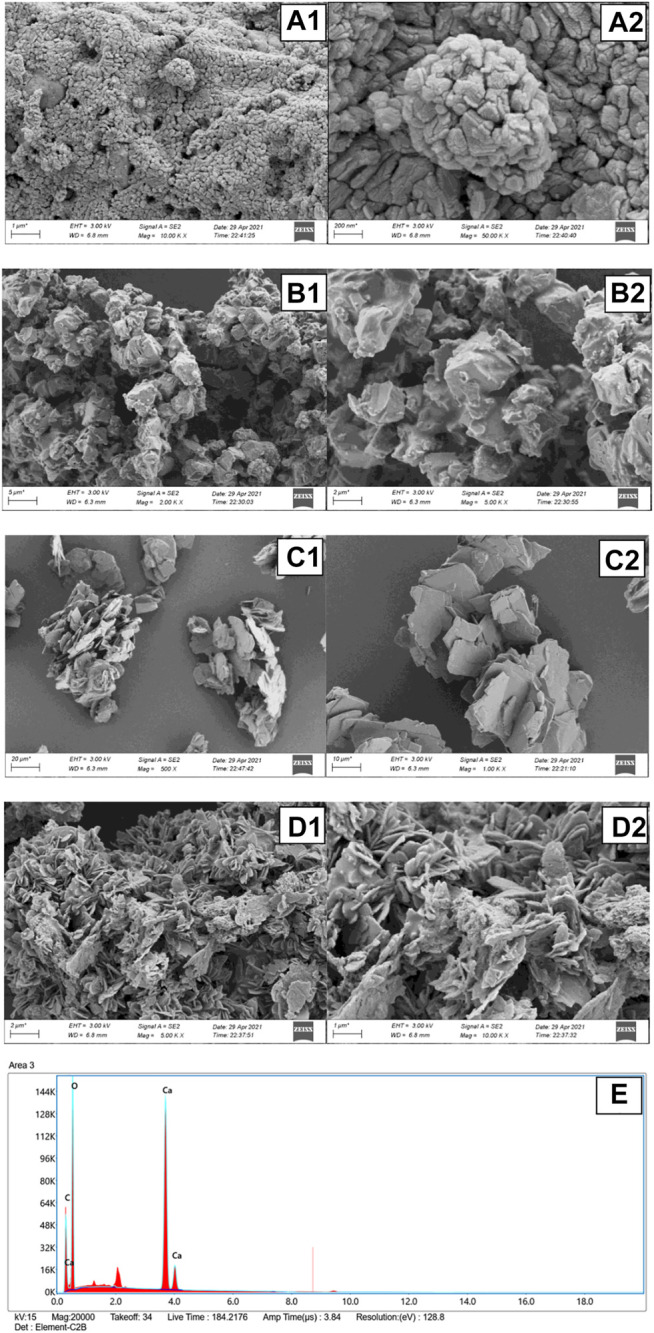
SEM images of the **(A)** fresh CaO, **(B)** CaDG, **(C)** CaG, and **(D)** CaG-E samples. **(E)** EDX profile of the CaG-E catalyst.

The morphologies of the fresh CaO, CaDG, CaG, and CaG-E catalysts were analysed by SEM (Figure 5). The fresh CaO surface contains irregularly shaped particles with considerable gaps and relatively large pores, which are related to the eggshell hollow structure. The enlarged image indicates the presence of cracks on the particle surface caused by the overflow of CO_2_ gas during the calcination process. The SEM image of CaDG (Figure 5B) shows the particles with sizes ranging from 7 to 10 μm and angular rock shapes, which is consistent with the results of previous studies conducted by Gupta et al.(Gupta and Rathod, 2018a) and Sánchez-Cantú et al.(Sánchez-Cantú et al., 2014). Figure 5C displays the SEM image of the synthesised CaG surface, which exhibits a single layer of irregular sheet structures. In Figure 5D, a large number of irregular lamellar structures (similar to CaG particles) are stacked on the CaG-E surface. This increases its specific surface area and facilitates the dissolution of CaG in glycerol during reuse; hence, CaG can be utilised as a homogeneous catalyst in the macrolactonisation process. In addition, EDX analysis was performed to determine the CaG-E composition (Figure 5E). The obtained spectrum shows that the analysed area contains 13 wt% carbon, 32 wt% oxygen, and 55 wt% calcium.

#### TEM and XPS Analyses of the CaG-E Catalyst

**FIGURE 6:**
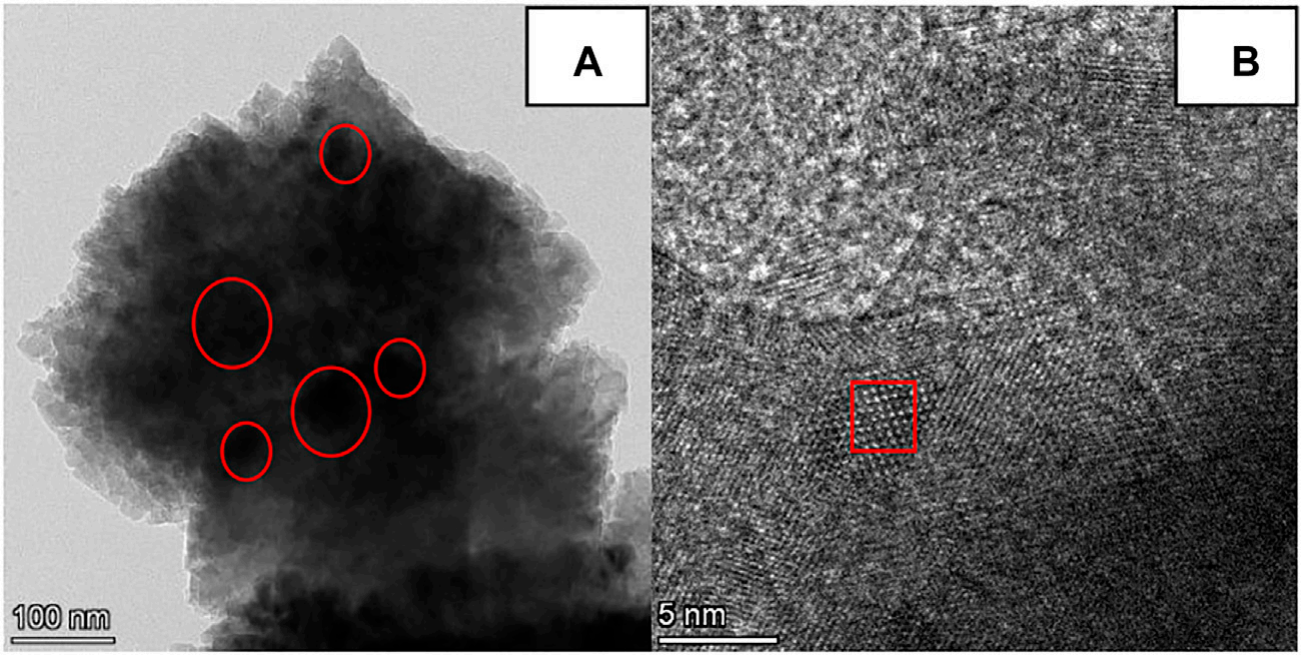
TEM images of the CaG-E catalyst at different magnifications, **(A)** 100 nm and **(B)** 5 nm.

The TEM images of the CaG-E sample are presented in Figure 6. Figure 6A shows the transparent edge with several black spots in the middle. The edge can be attributed to the CaG phase, and the black spots likely consist of partially calcined CaCO_3_ species. After increasing the TEM magnification, CaG lattice fringes were observed (Figure 6B). Therefore, CaG-E is composed of CaG and CaG-wrapped CaCO_3_ species.

**FIGURE 7:**
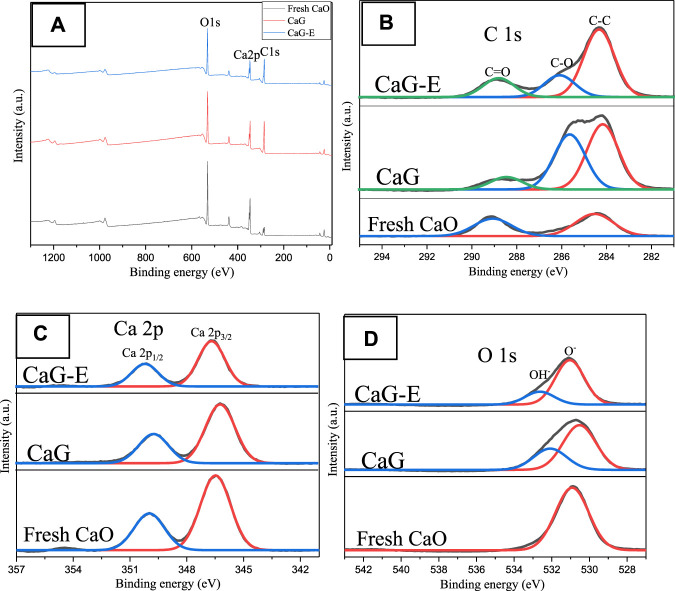
XPS **(A)** survey spectra of the CaG-E, CaG, and fresh CaO samples, and the corresponding core-level **(B)** C 1s, **(C)** Ca 2p, and **(D)** O 1s spectra.

The chemical compositions of the CaG-E, CaG, and fresh CaO samples were also determined by XPS. The survey scan spectrum contains three main peaks with binding energies corresponding to C 1s, Ca 2p, and O 1s species (Figure 7A). The C 1s core level spectrum exhibits two broad peaks consisting of the C–C (284.4 eV), C–O (286.1 eV), and O–C=O (288.8 eV) components. Compared with fresh CaO, both the CaG-E and CaG samples exhibit C–O peaks, and the intensities of the C–C peaks of the CaG-E and CaG samples are significantly larger than those of the other catalysts (Table 1), indicating the formation of a new compound on the CaG-E surface. The Ca 2p region exhibits two symmetric bands at 346.5 and 350.1 eV corresponding to a typical doublet of the Ca 2p_3/2_ and Ca 2p_1/2_ components with a separation of 3.6 eV. The Ca 2p_3/2_ peak has lower binding energies than those of CaO/calcite (347.1–347.7 eV), which suggests weaker interactions between Ca^2+^ and glyceroxide ions. The XPS profile of the CaG-E surface (Figure 7, CaG-E) shows a broad O 1s peak that can be deconvoluted into two components centred at 531.8 and 532.6 eV with areas of 37.7 and 10.9% (Table 1), which are attributed to OH^−^ and O^−^ groups, respectively (León-Reina et al., 2013). In Figure 6, the CaG-E and CaG peals are matched very well, suggesting that CaG is composed of glyceroxide anions, Ca^2+^ and OH^−^, and its likely chemical formula is HOCH_2_CHOHCH_2_OCaOH, which is consistent with the XRD spectra presented in Figure 4.

**TABLE 1:** XPS data obtained for CaG-E, CaG, and fresh CaO.

Catalyst	C (wt%)			Ca (wt%)		O (wt%)	
	C–C	C–O	O–C=O	Ca 2p_3/2_	Ca 2p_1/2_	O^−^	OH^−^
CaG-E	15.8	5.1	4.4	17.4	8.7	37.7	10.9
CaG	11.5	9.6	2.2	19.9	9.9	31.8	15.1
Fresh CaO	6.7	–	5.2	22.1	10.9	55.1	–

#### Basicity and Basic Strength Measurements Conducted by the Hammett Method

The basicity and basic strength of a catalyst play a vital role in the macrolactonisation reaction. The basic strengths of the catalysts prepared in this work were determined by the Hammett indicator method (Table 2). The basic strengths of CaDG and CaG were 9.8 < H_ ≤ 15.0 and 7.2 < H_ ≤ 15.0, respectively. An aqueous solution of CaG was used to measure its pH value equal to 13.0. The basic amounts of CaDG and CaG were 7.3 and 1.9 mmol g^−1^, respectively. The basicity and basic strength of CaG were lower than those of CaDG; however, the former compound produced a stronger catalytic effect because the high basicity of CaDG promoted the saponification of raw materials. Furthermore, the relatively low basicity of CaG increased it resistance to poisoning by ambient CO_2_ and H_2_O species and storage stability.

**TABLE 2:** Basicities and basic strengths of the CaDG and CaG catalysts.

Catalyst	Basic strength	Amount (mmol·g^−1^)
CaDG	9.8 < H_≤ 15.0	7.3
CaG	7.2 < H_≤ 15.0	1.9

#### Proposed Macrolactonisation Mechanism

**SCHEME 1:**
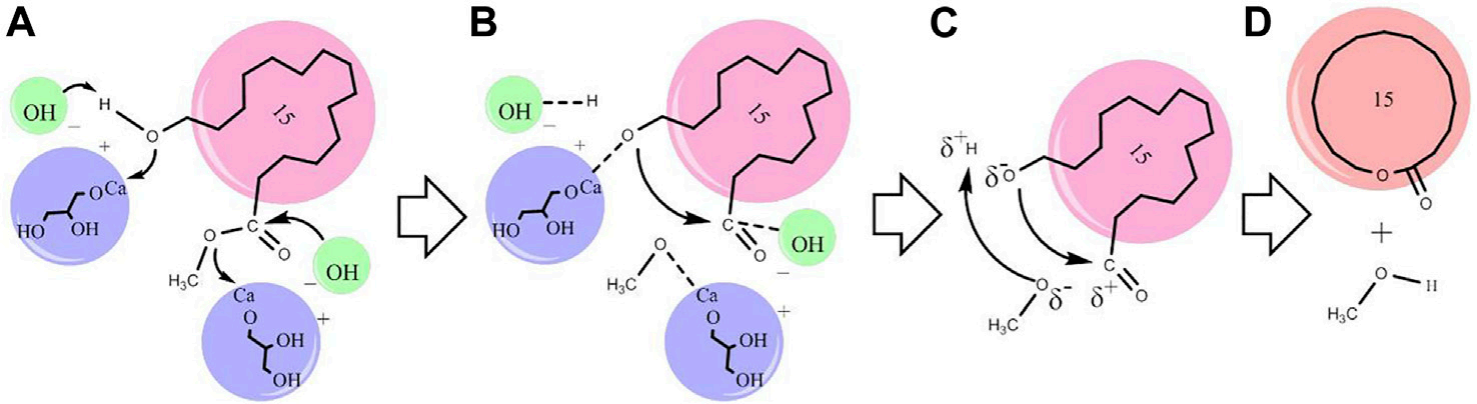
Proposed mechanism for the macrolactonisation of methyl 15-hydroxypentadecanoate using the CaG catalyst **(A–D)**.

The proposed macrolactonisation mechanism of methyl 15-hydroxypentadecanoate by CaG is presented in Scheme 1. In Here, CaG activates the carbon–oxygen bond of the terminal hydroxyl group and ester group of methyl 15-hydroxypentadecanoate. HOCH_2_CHOHCH_2_OCa^δ+^ and OH^δ−^ are the two active catalytic sites participating in the reaction. In step (A), ester and alcohol are adsorbed on these two neighbouring free catalytic sites. The adsorbed ester had formed an intermediate, while OH^δ−^ had extracted H^δ+^ and HOCH_2_CHOHCH_2_OCa^δ+^ adsorbed–O–CH_2_– from alcohol. By decreasing their electron density through the coordination with Ca atoms, C^δ+^ ions can undergo a nucleophilic attack by O^δ−^ anions. These two neighbouring adsorbed species react with each other in steps (B) and (C), leading to the formation of cyclopentadecanolide and methanol.

### Catalytic Activity

#### Effect of Catalyst Type on the Product Yield

**FIGURE 8:**
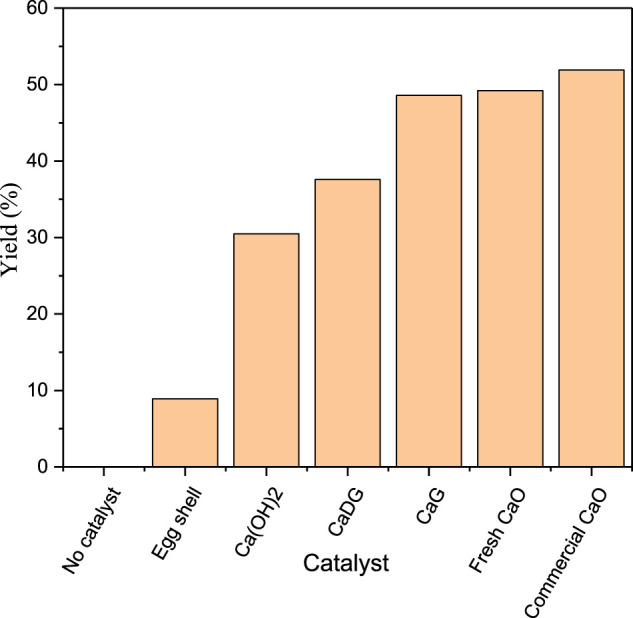
Effects of various catalysts for the macrolactonization of methyl 15-hydroxypentadecanoate on the product yield.

The catalytic effects of different catalysts on the macrolactonisation of methyl 15-Hydroxypentadecanoate are compared in Figure 8. The yield of cyclopentadecanolide is zero when no catalyst is added to the reaction mixture. Fresh CaO demonstrates a higher catalytic activity than those of eggshells, Ca(OH)_2_, and CaDG, while the catalytic activities of CaG and fresh CaO are very close. Finally, the catalytic effect of fresh CaO is slightly weaker than that of commercial CaO because eggshells contain small amounts of impurities such as magnesium carbonate in addition to CaCO_3_. After calcination, these impurities promote oxide formation and thus reduce the catalytic activity.

#### Effect of Reaction Conditions on the Product Yield

**FIGURE 9:**
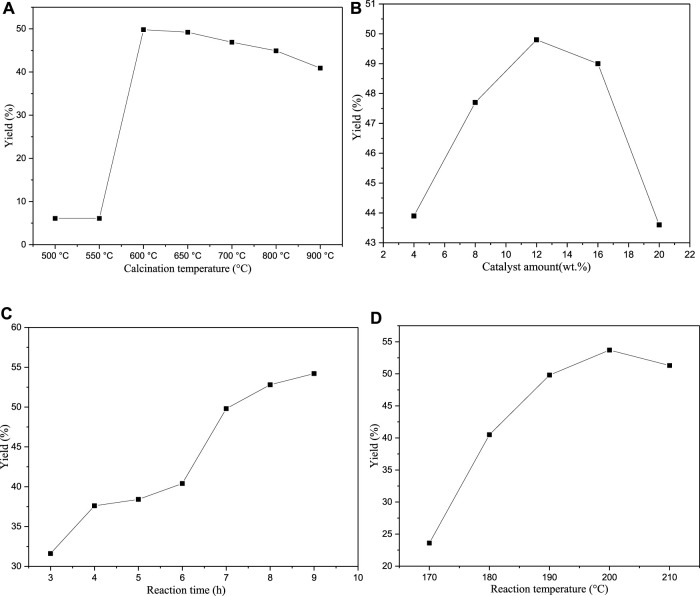
**(A–D)** Effects of various process parameters on the cyclopentadecanolide yield.

In this work, we optimised various process parameters, including the calcination temperatures of eggshells (500–900°C), catalyst amount (4–20 wt%), reaction time (3–8 h), and reaction temperature (170–210°C). The obtained results are shown in Figure 9. Note that to distil glycerol, the reaction must be performed under a vacuum of 2 mbar.

First, the effect of the calcination temperature of eggshells on the product yield was investigated. When the temperature is lower than 600°C, the cyclopentadecanolide yield cyclopentadecanolide is only 6.1%. Because the calcination temperature is too low, the CaCO_3_ phase in the eggshells is not decomposed into CaO. After the organic matter is carbonised, it forms a black solid layer on the catalyst surface, which ultimately deteriorates the catalytic effect. The yield rapidly increases to 49.8% at a roasting temperature of 600°C; however, the cyclopentadecanolide yield decreases with an increase in calcination temperature because the higher temperatures promote the CaCO_3_ conversion into CaO, increasing the CaO content. The glycerol-containing system produces a larger amount of colloidal Ca(OH)_2_, which inhibits the dissolution and diffusion of CaG species into glycerol and decreases the product yield. This trend is similar to that produced by the catalyst amount. When the amount of catalyst exceeds 12 wt%, the yield of cyclopentadecanolide decreases for the same reason.

Next, the effect of reaction time on the cyclopentadecanolide yield was investigated. Overall, the yield increases gradually with time. However, it rises very slowly when the reaction time exceeds 7 h, owing to the insufficiently high driving force for the reaction caused by the lower amounts of the raw materials. The best result was obtained at 7 h (49.8% yield) because only an additional 4.4% yield was achieved after the reaction time was increased by 2 h.

Finally, the impact of reaction temperature on the product yield was studied. As the reaction temperature increases, the yield first increases and then decreases after reaching 200°C. Because organic compounds are easily carbonised at high temperatures to increase the concentration of by-products, the saponification of the reactants in a strongly alkaline environment is likely to produce the corresponding salts. Based on these findings, 190°C was selected as the optimal reaction temperature. The related GC data indicated that the content of cyclopentadecanolide obtained in all reactions exceeded 95.0%.

### Performance of Recycled Catalysts

**FIGURE 10:**
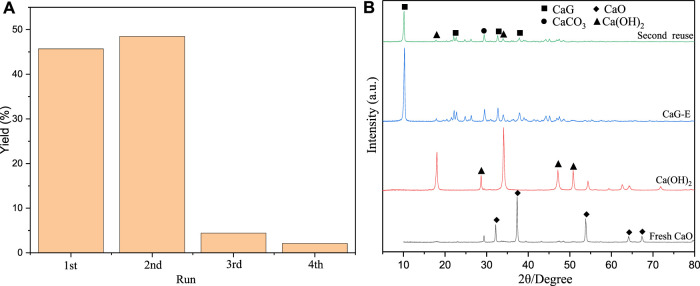
**(A)** Reusability of fresh CaO and **(B)** XRD patterns of the reused catalyst.

After the reaction was complete, the catalyst was separated by filtering, washed with ethanol, and dried under vacuum at 60°C overnight for the subsequent reuse and analysis (Figure 10). Interestingly, after the first reuse of fresh CaO, the yield of cyclopentadecanolide increased due to the formation of colloidal Ca(OH)_2_ on the catalyst surface after the first reaction with a large amount of wrapped CaG species. During the washing step conducted after recovering the catalyst from the first reaction medium, the colloidal forms of Ca(OH)_2_ were washed out, and a large amount of CaG was exposed to the surface, which was consistent with the SEM and TEM data. In addition, a large CaG amount was dissolved in glycerol to serve as a homogeneous basic catalyst; as a result, the product yield during the first repeated use was higher than that obtained for CaO. After the first repeated use of the catalyst, the yield dropped significantly due to the large loss of CaG. In the XRD pattern of the second recovered catalyst, the diffraction peak of CaG is significantly smaller, and the quality of the recovered catalyst is much lower than those of the fresh catalyst. After three repeated experiments, the cyclopentadecanolide yield was reduced to 2.1%.

### Proposed Mechanism of CaG Formation and Lixiviation

**SCHEME 2:**
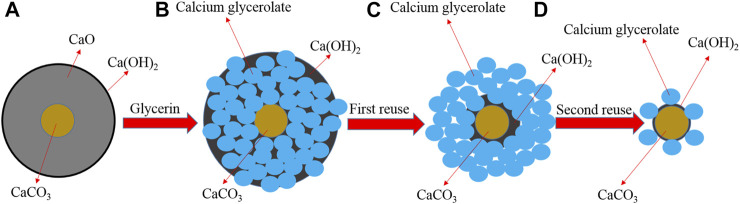
Proposed mechanism of CaG formation and lixiviation **(A–D)**.

From the obtained XRD results, a plausible CaG formation and lixiviation mechanism was proposed (Scheme 2). The fresh CaO catalyst is mainly composed of CaO, Ca(OH)_2_, and CaCO_3_ species (A). After fresh CaO enters the reaction, Ca(OH)_2_ colloids are preferentially formed. These colloids react with glycerol to produce CaG *in situ* (B), and CaCO_3_ nuclei are retained in the centres of catalyst particles. After the first washing and recovery step, CaG is exposed to the catalyst surface (C). When the catalyst is recycled for the second time, CaG is dissolved into glycerol, leaving mostly CaCO_3_ cores (D).

## Conclusion

In this study, the CaO catalyst prepared by calcining eggshell waste at a low temperature was used to catalyse the macrolactonisation of methyl 15-hydroxypentadecanoate. The characteristic changes in the catalyst morphology and composition after the reaction were characterised by XRD, SEM, TEM, and XPS, indicating that the main catalytically active component was CaG with the likely chemical formula HOCH_2_CHOHCH_2_OCaOH. Fresh CaO reacted with glycerine to form CaG, which acted as a homogeneous catalyst in the macrolactonisation reaction. The CaG-E catalyst was composed of CaG and CaCO_3_ species. The optimal macrolactonisation conditions determined by conducting single-factor experiments corresponded to the eggshell roasting temperature of 600°C, reaction temperature of 190°C, reaction time of 7 h, and catalyst amount of 12 wt%. Note that the utilised calcination temperature was only 600°C, which considerably decreased the energy consumption as compared with those of waste eggshell processes performed in other studies. Hence, the proposed catalyst can be obtained in large quantities by simply calcining eggshells in air at low temperatures. Its preparation method is simple, the reaction time is relatively short, and the product yield is high. The synthesised cyclopentadecanolactone is separated by reactive distillation. Because it is not soluble in glycerol, the obtained product does not require further purification, and its purity can exceed 95.0%. The method for the catalyst preparation from eggshell waste effectively recycles the waste, reduces the catalyst manufacturing cost, minimises the amount of contaminants, and makes the catalyst environmentally friendly. We used 15-tetracosenoic acid as a raw material, which may limit its industrial application. Therefore, finding other methods to synthesize methyl 15-hydroxypentadecanoate is our next work.

## Data Availability

The original contributions presented in the study are included in the article/supplementary material, further inquiries can be directed to the corresponding author.
